# Metastatic Squamous Cell Carcinoma to the Cervical Lymph Nodes From an Unknown Primary Cancer: Management in the HPV Era

**DOI:** 10.3389/fonc.2020.593164

**Published:** 2020-11-10

**Authors:** Francisco J. Civantos, Jan B. Vermorken, Jatin P. Shah, Alessandra Rinaldo, Carlos Suárez, Luiz P. Kowalski, Juan P. Rodrigo, Kerry Olsen, Primoz Strojan, Antti A. Mäkitie, Robert P. Takes, Remco de Bree, June Corry, Vinidh Paleri, Ashok R. Shaha, Dana M. Hartl, William Mendenhall, Cesare Piazza, Michael Hinni, K. Thomas Robbins, Ng Wai Tong, Alvaro Sanabria, Andres Coca-Pelaz, Johannes A. Langendijk, Juan Hernandez-Prera, Alfio Ferlito

**Affiliations:** ^1^Department of Otolaryngology, Sylvester Cancer Center, University of Miami, Miami, FL, United States; ^2^Department of Medical Oncology, Antwerp University Hospital, Edegem, Belgium; ^3^Department of Surgery, Head and Neck Service, Memorial Sloan Kettering Cancer Center, New York, NY, United States; ^4^University of Udine School of Medicine, Udine, Italy; ^5^Instituto de Investigación Sanitaria del Principado de Asturias, Oviedo, Spain; ^6^Department of Head and Neck Surgery and Otorhinolaryngology, A.C. Camargo Cancer Center, Sao Paolo, Brazil; ^7^Head and Neck Surgery Department, University of São Paulo Medical School, São Paulo, Brazil; ^8^Department of Otolaryngology, Hospital Universitario Central de Asturias, Oviedo, Spain; ^9^Department of Otorhinolaryngology, Mayo Clinic, Rochester, MN, United States; ^10^Department of Radiation Oncology Institute of Oncology, University of Ljubljana, Ljubljana, Slovenia; ^11^Department of Otorhinolaryngology—Head and Neck Surgery, University of Helsinki and HUS Helsinki University Hospital, Helsinki, Finland; ^12^Department of Otolaryngology/Head and Neck Surgery, Radboud University Medical Center, Nijmegen, Netherlands; ^13^Department of Head and Neck Surgical Oncology, University Medical Center Utrecht, Utrecht, Netherlands; ^14^Department of Medicine Division Radiation Oncology, St. Vincent’s Hospital, The University of Melbourne, Melbourne, VIC, Australia; ^15^Head and Neck Unit, The Royal Marsden Hospitals NHS Foundation Trust, London, United Kingdom; ^16^Division of Surgical Oncology, Gustave Roussy Cancer Center and Paris-Sud University, Paris, France; ^17^Department of Radiation Oncology, University of Florida College of Medicine, Gainesville, FL, United States; ^18^Department of Otorhinolaryngology, Maxillofacial and Thyroid Surgery, Fondazione IRCCS Istituto Nazionale dei Tumori, Milan, Italy; ^19^Department of Otolaryngology, Mayo Clinic, Phoenix, AZ, United States; ^20^Southern Illinois University School of Medicine, Department of Otolaryngology, Springfield, IL, United States; ^21^Department of Clinical Oncology, Pamela Youde Nethersole Eastern Hospital, Hong Kong, Hong Kong; ^22^Department of Surgery, School of Medicine, Hospital Universitario San Vicente Fundacion. CEXCA Centro de Excelencia en Enfermedades de Cabeza y Cuello, Universidad de Antioquia, Medellín, Colombia; ^23^Department of Radiation Oncology, University Medical Center Groningen, University of Groningen, Groningen, Netherlands; ^24^Department of Pathology, Moffitt Cancer Center, Tampa, FL, United States; ^25^International Head and Neck Scientific Group, Padua, Italy

**Keywords:** cervical adenopathy with unknown primary, HPV related head and neck cancer, non-HPV related head and neck cancer, molecular diagnoses occult primary, upper aerodigestive tract cancers, imaging head and neck cancer, transoral robotic surgery (TORS), transoral laser microlaryngoscopy (TLM)

## Abstract

**Background:**

Patients with metastases in the lymph nodes of the neck and no obvious primary tumor, neck cancer with unknown primary (NCUP), represent a management challenge. A majority of patients have metastatic squamous cell carcinoma (SCC), although other histologies do occur.

**Methods:**

We comprehensively reviewed the literature, compared available guidelines, and conferred with an international team of experts.

**Results:**

Positron emission tomography-computed tomography (PET-CT) and fine needle aspiration (FNA) under ultrasound guidance increase accuracy of diagnosis. Immunohistochemistry (IHC), determination of human papilloma virus (HPV) status, by p16 staining or by *in situ* hybridization (ISH), and next-generation gene sequencing can guide us regarding probable primary sites and tumor biology. Narrow Band Imaging (NBI) has been introduced for the early detection of subtle mucosal lesions. Direct laryngoscopy (DL) and tonsillectomy have long been procedures used in the search for a primary site. More recently, TransOral Robotic Surgery (TORS) or Transoral LASER Microsurgery (TLM) have been introduced for lingual tonsillectomy.

**Conclusions:**

New technologies have been developed which can better detect, diagnose, and treat occult primary tumors. Decisions regarding therapy are based on the primary tumor site (if discovered) and N stage. Options include neck dissection with or without postoperative adjuvant therapy, primary irradiation, or combined chemotherapy with irradiation. The preferred treatment of patients whose primary remains unidentified is controversial.

## Introduction

The patient with proven or suspected metastatic cancer in the cervical nodes and no evident primary cancer represents a unique challenge. The majority of these patients will have an occult squamous cell carcinoma of the upper aerodigestive tract, although infrequently other histological types and primary sites do occur. Identification of the primary site allows us to direct appropriate treatment strategies. This topic was well-reviewed by Strojan et al. (1, 2) in a two-part series in 2013. Our goal is to update the reader regarding the various innovations that have occurred in a time of increased incidence of HPV-related head and neck cancer.

## Definition of Metastatic Neck Carcinoma in the Neck With Unknown Primary

Many patients with cervical lymphatic metastases start out as “unknown primaries”, but most have primaries identified on careful physical examination, office endoscopy, and imaging (3). Only after such an evaluation can patients be categorized as neck cancer with an unknown primary (NCUP). We introduce more specific abbreviation NCUP rather than CUP, which is also commonly used for widespread systemic cancer metastases below the clavicles with unknown primary.

NCUP occurs in 1 to 7% of new head and neck cancer cases, and that percentage declines with the extensiveness of the search for a primary (4). After extensive endoscopic evaluation under general anesthesia, the percentage of unknown primary tumors decreases to less than 3%, (5, 6). Some have suggested that the incidence of patients with NCUP is increasing with the increasing numbers of HPV-related oropharyngeal cancers (OPCs) (7).

Metastatic lymph nodes in the parotid typically result from a cutaneous primary cancer and this represents a different discussion. We are focusing on patients who present with suspicious lymph nodes in levels I, II, III, and VA in the neck as described in the American Academy of Otolaryngology-Head and Neck Surgery (AAO-HNS) guidelines regarding standardized neck anatomic terminology (8). Squamous cell carcinoma metastases to lymph nodes in these anatomic levels can develop from skin cancer but are much more often from an upper aerodigestive tract primary. Other common histologies in the neck include well-differentiated thyroid malignancies, and nonsquamous malignancies originating in the skin, including melanoma, and Merkel cell carcinoma (high-grade neuroendocrine carcinoma), as well as salivary gland cancers (9).

Isolated supraclavicular nodes (level IV and VB), on the other hand, are either of thyroid origin or metastatic from primary sites below the clavicles, the classic “Virchow’s” node, which include gastrointestinal tract, urogenital tract, esophagus, biliary, liver, pancreas, lung, breast, and gynecological cancers (9–13). Even rarer malignant neck masses include soft tissue sarcomas and cervical metastases from central nervous system tumors (14, 15).

## Evaluation by the General Practitioner

Hayes Martin stated that “an adult patient who presents with a palpable lateral neck mass, whether solid or cystic, should be considered to have a metastatic lymph node until proven otherwise” (16, 17).

In the typical patient with NCUP, the lymph nodes, located in the upper part of the neck, are clearly abnormal in size, shape or consistency. The palpable mass may be firm or, if cystic, may have a tense or soft consistency. On careful questioning, the patient may have symptoms referable to a head and neck primary tumor, such as a sore throat when swallowing, ear pain, new nasal obstruction, voice change, etc. They also may have a history of tobacco and alcohol abuse or 10 or more lifetime sexual partners. The absence of suspicious history or symptoms does not, however, rule out cancer.

If a patient has a clinical presentation and imaging typical of lymphoma, with widespread adenopathy, sometimes exhibiting splenic, liver, bone marrow or lung involvement, and sometimes with “type B” constitutional symptoms (fever, weight loss and night sweats), this represents an appropriate clinical scenario for open cervical lymph node biopsy (18, 19). If not, it would be preferable to presume carcinoma initially and avoid open or even core biopsy as an initial test.

Since the 1940’s, the inappropriateness of premature excisional lymph node biopsy for patients with lateral neck masses has been well recognized (1, 20). The open biopsy itself does not make neck dissection necessary if chemoradiation is otherwise the standard treatment based on the primary tumor ultimately identified (21).

General physicians should have a strong understanding of the relationship of neck adenopathy to primary tumors of the upper aerodigestive tract, and a low threshold for referral to a head and neck specialist (22–24). Computed tomography (CT) of the neck with iodinated contrast or magnetic resonance imaging (MRI) with and without gadolinium contrast can be obtained while awaiting appointment with a head and neck specialist.

Ultrasound-guided FNA of the neck mass for cytology is also appropriate prior to referral, but core needle biopsy should be deferred until after evaluation by the specialist and complete head and neck physical examination including fiberoptic nasopharyngo-laryngoscopy (25).

## The Specialized Head and Neck Evaluation

### Clinical Evaluation

**Figure 1:**
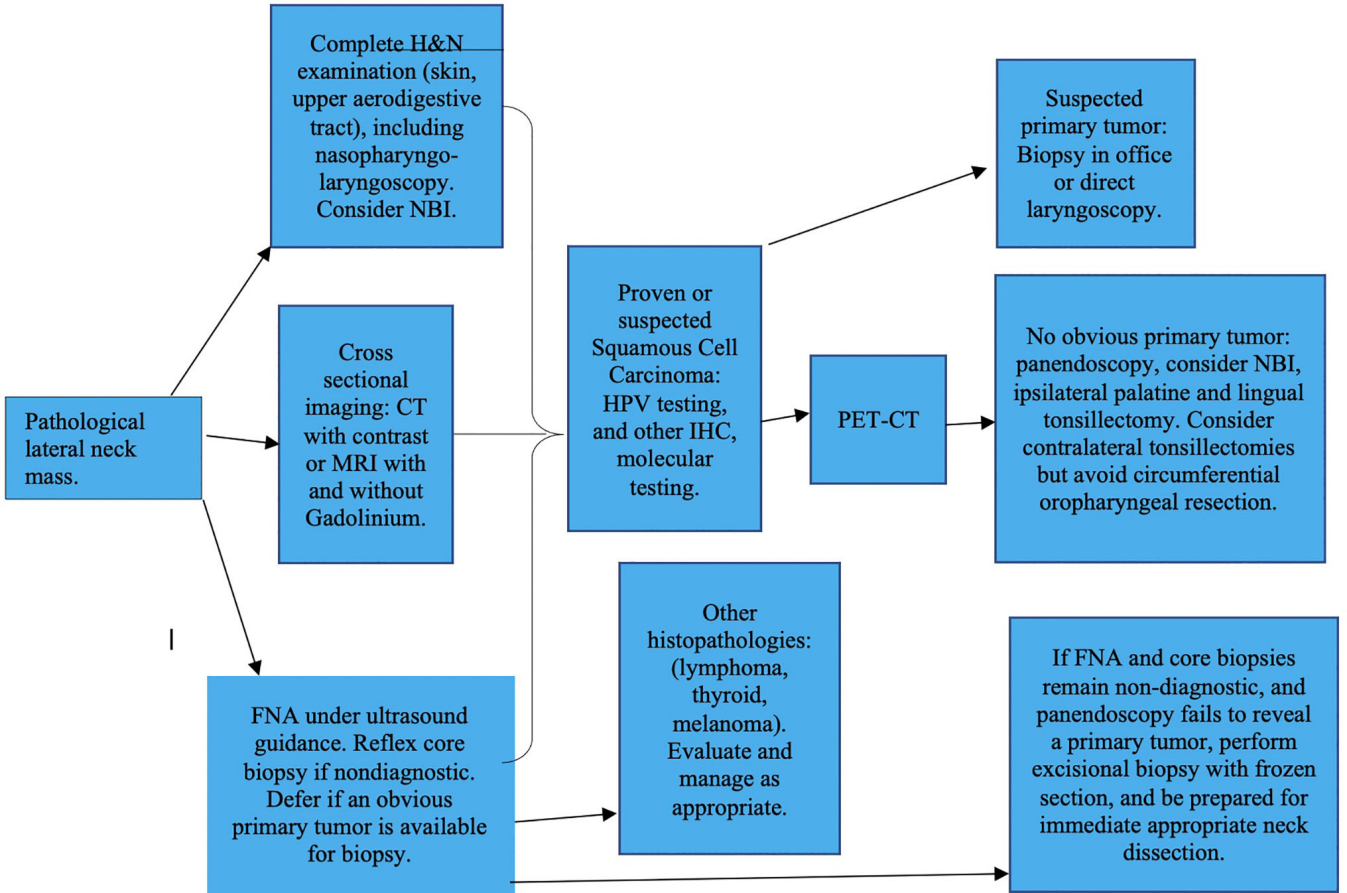
Evaluation of the adult patient presenting with pathological cervical adenopathy. H&N, Head and Neck; NBI, Narrow Band Imaging; CT, Computed Tomography; MRI, Magnetic Resonance Imaging; FNA, Fine Needle Aspiration; HPV, Human Papilloma Virus; IHC, Immunohistochemistry; PET-CT, Positron Emission Tomography-Computed Tomography.

Pertinent patient history and risk factors should be identified, followed by palpation of the neck, and meticulous physical examination of the upper aerodigestive tract including fiberoptic nasopharyngoscopy and laryngoscopy, palpation of the base of the tongue and tonsils, salivary glands and thyroid, inspection of the skin and evaluation of cranial nerve function. If a primary site is suspected, either office biopsy, or direct laryngoscopy and biopsy under general anesthesia should be scheduled. After this initial evaluation, imaging, if not already performed is requested (**Figure 1**). An ultrasound guided FNA of the neck mass is now obtained, if not already performed (9).

### Imaging

#### Ultrasound

Diagnostic ultrasound distinguishes solid from cystic masses, and provides details regarding intranodal anatomy and intrathyroidal anatomy. While optional for squamous cell carcinoma, in thyroid cancer it has a primary role in evaluating both thyroid nodules and lateral neck lymph nodes and in detecting pathological features in nodules and nodes that are not enlarged (26).

#### Cross-Sectional Imaging

CT with iodinated contrast or MRI of the neck extending from the skull base to the thoracic inlet is required. CT with contrast is typically the first test ordered. However, for patients with iodine allergy MRI with and without gadolinium will represent a reasonable alternative, and for patients with renal failure, non-contrasted MRI is the best option (25).

CT or MRI can delineate the anatomy of the cervical adenopathy, including the relationship of the abnormal nodes to vascular and visceral structures, and the presence of additional non-palpable but suspicious nodes, including possible adenopathy of parapharyngeal, retropharyngeal, paratracheal, and mediastinal areas. The presence of additional hidden nodes might point to a primary site. For example, retropharyngeal nodes would suggest a nasopharyngeal primary. Paratracheal adenopathy might suggest a thyroid or esophageal primary site. The imaging study may also directly image primary sites in the upper aerodigestive tract, detecting asymmetries of the mucosal surfaces toward which the specialist can direct their physical examination (25).

#### Metabolic Imaging: Positron Emission Tomography-Computed Tomography (PET-CT)

A significant development over the last 10 years has been the adoption of PET-CT as part of the initial imaging evaluation specifically for NCUP. Multiple publications suggest that PET-CT, while prone to false positives and limited accuracy overall, will direct the head and neck surgeon to a potential primary site in situations where other imaging modalities have failed (27–29). However, it is also well established that direct laryngoscopy under general anesthesia, particularly with the addition of tonsillectomy, will identify primary tumors in a significant percentage of patients with negative PET-CT (30). Sokoya et al. (30), in a 10-year retrospective study of 190 patients, reported that PET-CT had a sensitivity and negative predictive value (NPV) in detecting the primary site in unknown primary head and neck squamous cell carcinoma of 73.1% and 68.9%, respectively. Out of 103 patients whose PET-CT scans were negative for a primary tumor, 32 had primary tumors identified on direct laryngoscopy. Tonsillar cancer represented 56% of the primary tumors identified, 25% were in the base of tongue, 3% in the nasopharynx, and 3% in the hypopharynx. Multiple authors support the utility of PET-CT for NCUP as a guide prior to direct laryngoscopy, despite its limitations (27–30). In the meta-analysis by Zhu et al, PET-CT prior to endoscopy under general anesthesia allowed diagnosis of the primary site in 44% of cases with a sensitivity of 97% and a specificity of 68% (31). These results should be interpreted with caution, as the potential for detecting primary lesions through PET-CT largely depends on the extent and quality of the other diagnostic procedures performed.

## The Role of Fine Needle Aspiration Biopsy, Core Biopsy, and Open Biopsy

If a primary site is not identified on clinical examination, the next step will be FNA of the neck mass for cytological examination, preferably under ultrasound guidance (see **Figure 1** for workup of the suspicious neck mass). For patients with solid masses that harbor squamous cell carcinoma, FNA will be positive in 80% or more (32–34) and repeated FNA can yield an additional increment. With cystic neck masses the percentage is lower, but still, if ultrasound assisted and directed toward the most solid parts of the lesion, in the majority of patients with a suspicious neck mass, the FNA will correctly diagnose malignancy if present.

In those cases which are not squamous cell carcinomas, and in particular when a primary below the clavicles is suspected, sophisticated diagnostic approaches, including immunocytochemistry or immunohistochemistry(IHC) and molecular techniques are available to help direct a search for a primary site, based on the biopsy, and this is covered in more detail later.

With NCUP, the question of when to do an open biopsy of the neck mass becomes extremely important, since we have no guarantee that the primary will be located during the work up. FNA for cytology has a low but significant false negative rate, particularly for cystic lesions. False positives are much less common. Tandon et al. performed a review of 30 studies and then presented 2,702 aspirates from their own institution (35). Results were consistent for both parts of the study, and at their institution they reported sensitivity, specificity, positive predictive value (PPV), NPV, and accuracy rates of 89.5%, 98.5%, 97.3%, 94.0%, and 95.1%, respectively (35). These statistics are based on the precision of the diagnosis as given. For example, for thyroid nodules, a diagnosis of a follicular lesion would be considered positive and correct if it later proved to be a papillary cancer with follicular areas. A poorly differentiated malignancy might not be further classified on cytology. However, for squamous cell carcinomas the diagnosis was made exactly with a sensitivity of 92% (35).

It is therefore reasonable to start with FNA, since it is less likely to interfere with future surgery should that be the treatment of choice. Fine needles have also historically been favored as the initial procedure over cores, as traditional core biopsy instruments have ranged from 14 to 18 gauge in diameter, do not involve automatic suction devices, are performed from multiple directions, and can be quite traumatic. However FNA cytology is a triturated sample without tissue architecture, and a negative cytology of a suspicious mass is simply a failure to obtain diagnostic cells, and should not be considered benign (35).

The possibility of applying additional cytopathological and molecular techniques to the cytologic aspirate, to provide an estimate regarding the risk of malignancy relative to a benign branchial cleft cyst, is an area that is open for further study. If a few non-diagnostic cells are encountered, IHC or in-situ hybridization (ISH for HPV (36–38) Epstein Barr Virus (EBV), (39) P53 mutations (40), cytokeratin and other markers (41) can be performed, depending on the viral prevalence in the region and level of suspicion.

Thyroglobulin levels can be checked on fluid aspirated if thyroid malignancy is suspected. In a more unique approach, Nordemar et al. found that the presence of aneuploidy on DNA analysis predicted malignancy in 53% of malignant aspirates, and was never present in benign branchial cleft cysts (42). With further study, this and other sophisticated analyses of the fine needle aspirate could prove useful in providing an earlier diagnosis of malignancy.

HPV testing of initial cytology is important in the modern era, not only to help prove malignancy when material is scant, but also because of the importance of HPV status as a prognostic indicator and its potential for de-escalating the treatment approach. Although most patients will ultimately have histopathological tissue, either from a core, a primary site biopsy, or a neck dissection, early information regarding HPV status can direct the physicians toward the appropriate primary site and help them begin planning therapy. Daneshpajouhnejad et al. (43) used HPV-RNA ISH/P16 for HPV testing, a relatively new technique, and found a concordance rate of 88.9% between cytology and subsequent histopathology. HPV RNA significantly improved the accuracy of HPV testing of cytology specimens over P16 testing alone, which is sensitive but not very specific, as well as over the use of HPV-DNA ISH, which in previous publication was found to be technically challenging and less reliable (44).

Despite our ability to do more sophisticated testing, a non-diagnostic FNA can still occur. This can be due to low cellularity of the fluid within a cystic metastasis, the sampling of an area of necrosis, the sampling of peritumoral inflammation, excessive vascularity resulting in a bloody specimen, and other possible reasons (32). For cystic lesions, repeat FNA of a more solid component could be considered under ultrasound guidance (45). However, if the tumor is very vascular or very cystic or other technical issues exist that make repeating the aspiration futile, the head and neck surgeon may decide to proceed with a more invasive formal core biopsy procedure.

In order to improve the adequacy of FNA specimens, Allison et al. (46) recommended adding, at the time of the initial FNA, a single “small core” with a 20 gauge aspiration core device, as something close to a fine needle aspiration in terms of tissue trauma, but providing increased diagnostic yield, including more accurate IHC and ISH. Using this approach they demonstrated 100% accuracy of HPV IHC and in-situ hybridization (ISH) on the initial biopsy when surgical follow-up specimens were available.

Tabet et al. (32) specifically reviewed the management of cystic metastases with an unknown primary. In 135 cystic metastases of the neck, they found a high PPV of 92%, but sensitivity of only 59%. They discussed the role of ultrasound-guided core biopsy, in order to achieve a cancer diagnosis preoperatively, and confirmatory intraoperative frozen section at the time of open excisional biopsy performed as a first step with a plan for completion neck dissection if frozen section confirms positivity. Ultimately, 72% of their cystic neck masses in adults were proven malignant, despite presenting with clinical features common to branchial cleft cysts. Of these, 7.4% were papillary thyroid cancers, and 62% were squamous cell carcinomas. Among the squamous cell carcinomas, 17.8% had unknown primary tumors, a higher percentage than for solid metastases (32).

They recommended selective inclusion of formal core biopsies in the algorithm for lateral neck masses, particularly if cystic. This represents a change from the traditional approach, and has been adopted as a second line option by guidelines in both Europe and North America (9, 25, 47). For patients in whom lymphoma is highly suspected; core, incisional, or excisional biopsy, may be recommended as first step.

When fine needle aspiration fails to produce a diagnosis and cancer is strongly suspected, obtaining a positive core biopsy may provide the cancer diagnosis early, making it practically easier to justify the expense of a PET-CT before panendoscopy. This approach is supported by several large series showing that the incidence of clinically significant hematoma, infection, or seeding of the soft tissues with cancer from core biopsy is low (35–39, 48–58). At this point, National Cancer Center Network (NCCN) guidelines in the United States leave core biopsy as an option to confirm cancer before taking the patient to the operating room. If possible, the procedure should try to limit the number of directions from which core biopsies are obtained. Recently available, self-aspirating powered needles may reduce the already-low risk of tumor seeding, as tissue is extracted from a port without removal of the needle, allowing for multiple biopsies with a single pass. In addition, powered aspiration may reduce the presence of tumor fragments that could be dragged out at the time of needle removal (59).

Tabet et al. (32). found the sensitivity of core needle biopsy in cystic squamous cell carcinoma lymphatic metastases to be 87%. Frozen section at the time of excision had a 100% sensitivity for squamous cell carcinoma, though not for other types of malignancy. Given that core biopsy still results in 13% false negatives for cystic lesions, some patients will still need open biopsy to achieve the cancer diagnosis.

An argument in favor of core biopsy prior to endoscopy, particularly if the FNA cytology is completely non-diagnostic, is to detect diagnoses other than squamous cell carcinoma prior to going to the operating room, to avoid the rare, inadvertent misdiagnosis at frozen section leading to misguided treatment (56, 59).

For patients in whom a primary has not been located at panendoscopy, and for whom non-surgical therapy is planned, we would strongly encourage a core biopsy to achieve an accurate histological diagnosis, rather than embarking on a course of chemotherapy and radiation based entirely on cytology. An exception may be made if PCR for HPV or EBV is positive on cytology, and the cytology appears unequivocal. However, it is easy to be fooled, and when the histopathology is not available, we do not know if an infrequent finding is present that can confound cytology. The “gold standard” is to enter radiation or chemoradiation treatment based on histology rather than cytology. This allows us to identify histologic subgroups that might fall under the cytologic umbrella of “squamous cell carcinoma” (i.e. nasopharyngeal-type or “lymphoepithelioma”, basaloid, spindle cell, papillary, undifferentiated, adenoid squamous, etc.), but have specific clinical features that make a difference, as well as related pathologies (i.e. mucoepidermoid carcinoma) that can be misread as squamous cell carcinoma on cytology. More IHC and molecular tests generally are more accurate in histopathologic specimens (32). Excisional biopsy of a mass or node, without completion neck dissection, offers no therapeutic advantage over a less-invasive positive core biopsy, as the patient needs the same dose of radiation.

## Advanced Pathological and Molecular Diagnosis for Identifying Primary Sites

After reviewing imaging, and before endoscopic procedures, our next step should be more detailed evaluation of neck pathology. Twenty years ago, the first reports strongly associating a new, less aggressive variant of OPC with human papilloma virus (HPV) infection were published (57). Since then, as smoking rates decreased, HPV-related OPC, characterized by the presence of high-risk HPV deoxyribonucleic acid (DNA) detected by ISH and/or real time reverse transcription polymerase chain reaction (RT-PCR), has increased in incidence relative to the tobacco-associated form of OPC which is less restricted to the oropharyngeal subsite. IHC for p16 serves as a surrogate marker for HPV-related cancer, but can yield false positive results, and should be confirmed by HPV detection when we encounter tumors that do not have either the classic basaloid-like histology or the typical anatomic location in the lingual or palatine tonsils. Histologically, HPV-related cancers tend to be exophytic and basaloid in appearance while p16-negative cancers are frequently infiltrative and are often associated with P53 mutations. There is a heterogeneity of phenotypes within the p16-negative group, and we will likely refine our understanding regarding the different presentations of p16-negative cancers as research progresses in coming years (36, 38, 57, 60). Cancers outside of Waldeyer’s ring, including oral cavity, larynx and hypopharynx, are usually p16-negative. According to the guidelines of the College of American Pathologists, if a squamous cell carcinoma is p16-positive without the typical basaloid-like histology or in an anatomic location other than palatine or lingual tonsil, HPV DNA detection with ISH or RT-PCR are required to categorize the patient as HPV related head and neck cancer. In addition, if working with a cytological sample on a suspected HPV-related cancer, HPV DNA detection with ISH or RT-PCR must be performed, as false negative p16 results may be encountered frequently in cytological specimens (36, 57, 60).

In North America and Western Europe in recent years the majority of oropharyngeal cancers are HPV-related (70%) and the incidence of oropharyngeal cancer has increased significantly. The percentage of other (non-OPC) anatomic subsites that are HPV related are much smaller and may not carry the same implications of improved prognosis (38, 61–64).

The diagnosis of an HPV related tumor may be suspected based on the IHC, the presence of cystic metastases, or the identification of basaloid cytology or histology, and IHC stains, particularly for p16. Once this diagnosis is suspected or proven, it immediately directs the head and neck surgeon toward the oropharynx at the time of either office examination or direct laryngoscopy.

The presence of p16-negative cancer in the node on the other hand, will lead the head and neck surgeon to consider the entire upper aerodigestive tract at risk. The lymphoid tissue of Waldeyer’s ring, because it is a site of frequent anatomic irregularity, remains a site to be sampled, but the head and neck surgeon will also give extra attention to the larynx (especially to the supraglottis), nasal cavity, oral cavity (with special attention to gingiva and oral tongue), hypopharynx, and cervical esophagus.

While less frequently tested in Europe and North America, in the case of clinical suspicion of nasopharyngeal origin or nasopharyngeal-type histology, or inability to find a primary tumor after panendoscopy, testing for EBV in the neck specimen is an option (65, 66). Luo et al. looked at the distribution of located primary sites in EBV positive NCUP, and anatomic distribution of adenopathy in 269 patients. The distribution of primary sites included nasopharynx (51.7%), salivary gland (24.5%), lung (7.8%), oropharynx (3.3%), nasal cavity/maxillary (3.3%), oral cavity (2.2%), orbit (1.1%), and liver (0.4%). The rare EBV positive primary lung and liver cancers that metastasized to the neck, always presented in level IV. Level V metastases were infrequent from sources other than nasopharynx (67).

In Southern China, the Middle East and North Africa, EBV testing is more important than HPV. The location of adenopathy in the posterior triangle or retropharyngeal nodes might be a clue in such a situation, but solitary level II nodes can occur, as level II nodes are actually the second most frequent site (70%) from a nasopharyngeal primary after retropharyngeal nodes (67, 68). Testing for EBV involves in-situ hybridization for EBV encoding region (EBER), a test which has been well validated (65–68).

If a cancer in the neck is so poorly differentiated that the pathologist is not able to assign squamous origin using a cytokeratin stain, a broader panel of immunohistochemical stains becomes important to differentiate squamous cell carcinoma from lymphoma, adenocarcinoma (thyroid or other), melanoma, neuroendocrine carcinoma, medullary thyroid cancer, salivary histologies, etc. Various immunohistochemical markers such as thyroglobulin, calcitonin, thyroid transcription factor-1 (TTF), Paired-box gene 8 (PAX8), S100 protein, human melanoma black 45 (HMB 45) can be used (69). Extremely poorly differentiated lymphomas can express some keratin and lead to initial misdiagnosis, and flow cytometry and IHC panels must be directed toward their exclusion. Flow cytometry and IHC for CD20 and PAX-5, among other B cell markers, can be very helpful, along with heavy chain gene rearrangement by RT-PCR to demonstrate clonality (70).

In geographic regions where cutaneous squamous cell carcinoma is frequent, diagnostic evaluation of metastatic carcinoma in lymph nodes of levels I to V, can be difficult. Even with a history of skin cancer, patients will have to undergo endoscopic evaluations and biopsies to rule out an upper aerodigestive tract primary. Furthermore, cutaneous squamous cell carcinoma can frequently be p16 positive, without any implications regarding viral etiology or prognosis, further confusing the picture (71, 72).

Once histopathological tissue is available from the lymph nodes, new clinically available technologies for “Next Generation Sequencing” (NGS), i.e. genetic profiling of (DNA) sequences, can be obtained, and can produce gene profiles associated with ultraviolet light damage which are more consistent with skin cancer, or conversely, profiles consistent with upper aerodigestive tract cancer (73). This knowledge may affect decisions regarding irradiation of the upper aerodigestive tract or the contralateral side of the neck postoperatively. As time progresses NGS will probably settle many additional diagnostic questions where histology and immunohistochemistry fall short. Ultimately sequences for HPV-related mutations, EBV-related mutations, thyroid cancer associated molecular profiles, etc. could all prove useful (73).

The different DNA sequences encountered in skin cancer versus upper aerodigestive tract cancer is only one example of molecular profiling of tumors in order to identify their origin. These technologies have been extensively used in the development of “liquid biopsies”, evaluating circulating cancer cells (CTCs) and cell-free DNA (ctDNA) in a blood sample, which are well-described for widespread systemic cancer metastases with unknown primary (CUP), and can involve not only gene profiling but also gene microarrays (a different genetic sequencing technology from NGS), and microRNA and DNA methylation analysis (41, 74–77). Molecular signatures produced through these technologies can predict the probable primary site throughout the body with accuracies of 80-95% (74, 77). Whether these technologies could be further developed to distinguish between closely related primary sites within the head and neck for NCUP remains to be determined.

## Endoscopic Procedures: Panendoscopy, Narrow Band Imaging, Transoral Robotic Surgery, and Transoral Laser Microsurgery

In NCUP of levels I, II, III, and VA of the neck, the next step in the traditional algorithm is the panendoscopy or “triple endoscopy”, including direct laryngoscopy, rigid or flexible bronchoscopy, and rigid or flexible esophagoscopy (78). Due to the exceedingly low incidence of clinically occult primary in the lung or esophagus with a metastatic node in the neck, many centers have now abandoned the practice of bronchoscopy and esophagoscopy in the search of the unknown primary. Bronchoscopy and esophagoscopy as endoscopic screening for second primaries, not causative of the neck mass, also remains controversial, but has its advocates (79–83). On the other hand direct laryngoscopy and careful endoscopy of the nasopharynx is clearly warranted. Examination under anesthesia is far superior to the office examination at identifying a primary tumor, because of relaxation of the pharyngeal musculature and ability to palpate base of tongue, tonsils and nasopharynx. Any firm nodularity, or bleeding on palpation requires biopsy of these suspicious areas (79, 84, 85).

The literature prior to the era of HPV-related cancer showed increased survival associated with the initial identification of the primary tumor in patients with NCUP (86–88). However more recent publications have not been able to show statistically improved survival rates, although other benefits occur with identification of primary tumors, such as precision in planning radiation ports (89).

Nonetheless, it is logical to do all we can to identify a primary site. This is tantamount to establishing the correct diagnosis, as NCUP is a “wastebasket category” that will potentially include cutaneous, hypopharyngeal, nasopharyngeal, oral cavity, laryngeal, and oropharyngeal cancers, all of which have different biological behaviors and would benefit from different treatment algorithms.

Beyond panendoscopy and biopsy of visible and palpable lesions, the first attempt to more extensively sample the upper aerodigestive tract, was the practice of performing random biopsies of the nasopharynx, oropharynx, hypopharynx and tongue base in the absence of abnormal endoscopic findings. These were considered standard for many years and could fortuitously yield unexpected positive results in rare cases. The yield on such biopsies however was very low, and this practice has largely been abandoned (90). Such random biopsies are no longer included in the NCCN Guidelines, and where tonsillectomy and lingual tonsil biopsies are advised as well as directed biopsies based on endoscopic findings (9).

The obvious limitations of random biopsies led to the development of technologies designed to help the head and neck surgeon identify the proper site to biopsy in order to find a primary tumor that is imperceptible to the eye. The most obvious first step is carefully looking at all of the mucosa under microscopic magnification and searching for areas of subtle leukoplakia, erythroplasia, roughness, or minimal papillomatous tissue. More powerful optical enhancement technologies have also been developed. The most proven of these is narrow band imaging (NBI), which works by restricting light to two spectral wavelengths, the blue (400-430nm) and green (525-555 nm). The blue enhances capillaries and green enhances deeper blood vessels, and the overall effect makes lesions, whether dysplastic or early malignancy, easier to detect. It can be used during office fiberoptic nasopharyngoscopy or at the time of panendoscopy under anesthesia (91).

In combination with white light imaging, NBI has high sensitivity and negative predictive values. In one systematic review, the sensitivity and specificity for NBI in NCUP setting was 74% (range, 58% to 87%) and 86% (range, 76% to 93%), respectively. This high accuracy was confirmed by the area under the curve of >0.9, suggesting that NBI is an excellent tool for localizing the mucosal primary in patients with NCUP (92). A more recent systematic review confirmed this accuracy, with a pooled sensitivity and specificity in patients with NCUP of 0.83 (99% CI, 0.54–0.95) and 0.88 (99% CI, 0.55–0.97), respectively. The pooled diagnostic odds ratio (DOR) was 82.15 (99% CI, 7.06–955) and the overall detection rate of NBI was 0.35 (99% CI, 0.18–0.53), which allowed localization of the primary tumor in 61 out of 169 patients (93). These studies suggest that NBI could have considerable diagnostic value in NCUP, increasing the likelihood of finding the mucosal lesion that is responsible for regional metastatic spread to the neck. Therefore, this non-invasive adjunct to the conventional fiberoptic examination should be considered for routine use in these patients.

There has also been a movement toward removing normal appearing tonsillar tissue as a means of achieving a diagnosis of a primary tumor. First unilateral and then bilateral palatine tonsillectomy became standard for NCUP in the 1980’s and 1990’s, which was proven to increase the identification of primary tumor sites in a significant percentage of patients, while adding little additional long-term morbidity (89, 94, 95). Interestingly, approximately 10% of tonsillar primaries are found in the tonsil contralateral to the lymphatic metastases (96).

Unlike the palatine tonsils, lingual tonsil removal was not a common operation traditionally. The lingual tonsils are not frequently chronically infected, are not directly accessible transorally, require access at a 30-degree upward angle, and are less encapsulated than the palatine tonsils. Lingual tonsillectomy was not widely performed for non-neoplastic indications. As the surgical robot became available, different medical specialties started to incorporate its use. In head and neck surgery the da Vinci surgical robot, manufactured by the Intuitive Surgical, was first applied to the transoral resection of cancers of the oropharynx (TORS) (97).

Mehta et al. in 2013 first reported endoscopic bilateral lingual tonsillectomy with the assistance of the surgical robot in NCUP. They performed this procedure on 10 patients who had already had direct laryngoscopy, directed or random biopsies, and palatine tonsillectomies. Primary tumors were identified in the lingual tonsil tissue in 9 of the 10 with a mean diameter of 0.9 centimeters (98). In the same year Nagel et al. published the results of 36 lingual tonsillectomies in NCUP using laser microsurgery with identification of the primary tumor in 86% (99).

Multiple other authors have confirmed the effectiveness of robotic lingual tonsillectomy at locating primary tumors (90, 98, 100). Ryan et al. (89)reviewed the records of 110 patients diagnosed with NCUP. Direct laryngoscopy identified a primary tumor in 31% of patients. Forty-seven patients underwent palatine tonsillectomy, which identified 17 primaries (36%), yielding a cumulative primary tumor identification of 51/110 (46%). Finally, 14 patients underwent TORS lingual tonsillectomy, which identified eight primaries (57%), resulting in a cumulative identification of 59/110 (53%). The detection rate increased from 44% to 66% after the addition of lingual tonsillectomy.

The use of transoral laser microsurgery (TLM) to achieve the same end, using a combination of microscope and telescopes, has also been described by multiple other authors (101–103).Regardless of the technique chosen, palatine and lingual tonsillectomy will locate a primary tumor in approximately 70% of patients with NCUP who have completely negative office evaluations and imaging, including PET-CT, particularly in the HPV+ setting (102).

Technically, it is important to preserve uninvolved non-lymphoid mucosa between the tonsillar tissue and avoid a circumferential wound, in order to prevent oropharyngeal stenosis. Thus, patients have done well functionally as long as an area, often in the contralateral lingual tonsil and glossopharyngeal sulcus, is sampled but not removed en bloc. The surgical robot, is a useful instrument which allows the surgeon to work in a tight space controlling the small “hands” of the instrument by remote control with cautery, graspers, etc. When the robot is not available, we have found it helpful to work two-handed with the FK retractor, and an assistant holding a 30-degree telescope to provide the angle needed to visualize the lingual tonsil well, and this can be complementary to the microscope.

Although no literature discusses this formally, if a patient with NCUP has a significant adenoid pad, unusual in the adult age group, adenoidectomy should be considered under the same logic that leads us to do palatine and lingual tonsillectomies. It should also be mentioned that, although the likelihood of an oropharyngeal primary is higher in HPV positive patients, the algorithm for management of the primary in the neck, in terms of imaging, endoscopies, and removal of lymphoid tissue, as illustrated in **Figure 1**, is similar for all squamous cell carcinomas in the cervical lymphatics, regardless of HPV status.

The combination of wounds in both tonsils, across the tongue base, and in the nasopharynx, adds additional risk of bleeding and leads to odynophagia in the short term. Since the cancer is more likely to be ipsilateral than contralateral, it is unclear when and how to perform all of these staging procedures, allowing for the maximal staging benefit while minimizing risk and discomfort. We can start with panendoscopy, and biopsy any area that stands out as irregular or that bleeds easily. The next step would be to perform palatine tonsillectomies, and obtain frozen section, followed by ipsilateral lingual tonsillectomy and frozen section, and then contralateral lingual tonsillectomy. If a primary has not been identified, we can now consider sampling any available lymphoid tissue in the nasopharynx. With such an approach, the majority of primary tumors should be identified. It needs to be acknowledged that frozen section is not as sensitive as definitive histology and that the primary may still not be seen until final pathology.

To reduce or avoid the use of frozen section, the procedure can, alternatively, be divided, with panendoscopy and palatine tonsillectomy at one intervention and lingual tonsillectomy at a second one. This approach produces throat pain and risk of bleeding for the patients twice rather than once. Some propose it may reduce the risk of oropharyngeal stenosis. The risk-benefit ratio of performing palatine tonsillectomy at the same time as lingual tonsillectomy versus in a staged fashion has not been formally studied in a randomized trial.

## Undiscovered Primaries Remaining After Extensive Endoscopies and Endoscopic Resections: Are they Unique?

One might ask where the primary tumor is hidden in a patient who has undergone careful endoscopy with optical enhancement, and has had all of their pharyngeal lymphatic tissue removed. The incidence of subsequent development of clinically evident primary tumors in patients who receive neck dissection for NCUP and are observed without radiation is relatively low, between 10 and 30% in an era where palatine tonsillectomies and lingual tonsillectomies were not done (104, 105).Yet in theory, since no treatment was directed to the mucosal primary, a clinically progressive throat cancer should have become evident over time in 100% of those patients. This has led some to propose that regression of the primary tumor was occurring in occasional cases.

Tumor regression can infrequently occur for other tumor types that are known to be very immune sensitive, such as cutaneous Merkel cell carcinoma and melanoma, and there are actually hundreds of case reports of regressed cancers, including some squamous cell carcinomas (106, 107). However, the experience in patients with clinically evident squamous cell carcinoma would indicate that once there is a measurable primary cancer, it is extremely rare for this to regress.

On the other hand, Califano et al. in 1999 performed a very interesting study that may provide some insight. Their group performed microsatellite analysis on metastatic adenopathy and benign surveillance biopsies from 18 patients with NCUP. In 10 of the 18 patients, at least one benign biopsy specimen from sites at risk for occult primary tumor demonstrated a pattern of genetic alterations identical to that present in the lymph node metastases. Thus, while the tissue was phenotypically benign, it was “genetically malignant” (108).

Two of their patients subsequently grew cancers in the same mucosal region. They proposed that phenotypically normal mucosa, perhaps with foci of phenotypic cancer so small they could not get picked up on biopsy, had sufficient mutations to permit those cells to metastasize to lymph nodes, but not to behave in a locally proliferative or invasive manner at the primary site. He proposed that mucosal biopsies could be evaluated genetically and that areas with genetic signatures that matched the cancer could be targeted for radiation. Obviously, this concept would need to be validated, but clearly this would represent a much more sophisticated level of evaluation, and merits investigation (108).

## Treatment

Treatment of NCUP is based on the primary tumor site, if identified, and on the N stage of the neck disease, as it would be for any other head and neck cancer. The nuances of navigating treatment in the small group of patients in whom the primary tumor remains unknown after an extensive search were covered in great detail by Strojan et al. in 2013 who dedicated an entire review in their two part publication to this subject (2). Since that publication 7 years ago the principal change that has occurred in North America and Europe is the routine testing of all OPCs, and sometimes other anatomic subsites as well, for p16 IHC, and sometimes for HPV DNA or mRNA ISH or RT-PCR, in order to categorize tumors as HPV-related or not.

The first step in planning treatment always involves a hypothesis regarding the probable occult primary site. The first clue used is the distribution of the adenopathy by anatomic levels. One would expect this distribution to vary by geographic region depending on the prevalence of cutaneous malignancy, which varies greatly by ethnicity and skin type, as well as by the prevalence of EBV and HPV related upper aerodigestive tract cancers. In general, adenopathy in NCUP is most commonly in level II, which suggests primary sites in the oropharynx, hypopharynx, or supraglottic larynx, although isolated level II metastases can also occur with oral cavity cancer and nasopharyngeal cancer. NCUP with isolated level I (submandibular and submental) adenopathy is less frequent, and points to oral cavity and skin primary sites. As part of a study targeting level I, Ozer et al. in 2010 reported that level I adenopathy occurred in 2 (11.1%) of 18 patients with NCUP (109). The rest of their level I nodes were distributed among known oral cavity (19.1%), oropharynx (9.8%), and larynx (4.4%) cases, and none were observed with hypopharyngeal cancer (0%). Oral cavity cancer in particular went to level I in 55% followed by level II (20%) and level III (19%), and level IV and V in only 3% each (and generally the latter were in the presence of extensive adenopathy in levels I, II and III). OPC patients only had level I adenopathy in the presence of extensive adenopathy in other levels. One might anticipate a lower incidence of level I NCUP in regions that have lower rates of cutaneous malignancy (110).

Similarly, the presence of level V adenopathy will lead to consideration of primary tumors of the nasopharynx or posterior scalp (68, 111). Given the predominance of EBV-negative NCUP in levels II and III, our discussion regarding treatment will be focused on this group.

It is well documented that the rate of appearance of a clinically evident primary tumor in the upper aerodigestive tract during follow-up surveillance after treatment of NCUP is low (112, 113). With neck dissection and close observation, while primary emergence still does not occur in the vast majority of patients, there is a somewhat higher rate of development of clinically evident primary tumors in the upper aerodigestive tract compared to patients who receive radiation to the upper aerodigestive tract. These numbers range widely, possibly depending on factors such as the completeness of the endoscopic evaluations to search for a primary, the prevalence of EBV and HPV related cancers in the region, and the prevalence of cutaneous malignancy (112, 113). Primary site emergence rates can range from 2% to 50% with most recent reports being on the lower end of this spectrum (112, 114). These data are hard to interpret as it is difficult to know, for each center, how aggressive the initial search for a primary tumor was. For centers reporting lower rates, these approximate the rate of expected development of new primary tumors in patients cured from known primary tumor, and there is a question as to whether we are actually just detecting new primary tumors in the at-risk mucosal field (2).

Interestingly recent reports regarding unilateral neck radiation without mucosal fields have reported extremely low primary emergence. Takes et al. for a population that was primarily HPV negative, reported equivalent survival and no primary site emergence after unilateral irradiation to the neck in 42 patients with low volume unilateral NCUP (115). Others have reported similar results in HPV negative populations in recent years (114). Strojan et al. found no significant difference when they compared the risk of metachronous second primary tumors in patients with known primaries, and the risk of appearance of a mucosal primary in NCUP patients who were diagnosed during the same time span and were postoperatively irradiated either with involved-field or extended-field technique (116). Certainly, with HPV-related cancer that has undergone tonsillectomies and partial lingual tonsillectomy, it appears even less likely for a primary to emerge in a delayed fashion (117).

The reasons that primary emergence appears to be much less frequent in this setting is unclear, but we can conjecture that with primary neck radiation we are likely delivering enough dose to the at-risk mucosal sites, particularly the ipsilateral tonsil and tongue base, to achieve occult primary control (118). This seems unlikely to completely explain the reduced rate of primary emergence, as certainly some contralateral and out-of-field primary sites would be expected to emerge. A rising incidence of squamous cell carcinoma of the skin and more rigorous pretreatment evaluation of pharyngolaryngeal mucosal sites may also be factors (110, 111).

In 2018 the American Joint Committee on Cancer (AJCC) updated its staging system for HPV-related OPCs, (119) which makes discussion based on stage more difficult. However, for low volumes of neck disease, as advocated by Strojan et al., and others (2, 112, 120), it is clear that one option is radiation alone, preferably after core biopsy for histology. After unilateral neck irradiation of nodal disease, without attempting to comprehensively treat mucosa aggressively, but rather selectively treating highest risk areas, cure rates of 92% for early disease are obtained (2, 105). Treatment volumes are targeted based on tumor volume, anatomic levels of neck involvement, and HPV or EBV status. Radiation typically involves intensity modulated radiation therapy (IMRT) or 3D conformal radiation to reduce dose to critical structures with doses of 66 to 70 Gy, 2–2.2 Gy per fraction, on weekdays over approximately 6 to 7 weeks. For lower risk anatomic areas lower doses are used between 44 and 63 Gy (9). Another approach is to treat as one would for a T1 oropharyngeal SCC, because most of the NCUP are likely located in the tonsil and tongue base. This involves irradiation to the oropharynx, retropharyngeal nodes, and both sides of the neck. The main disadvantage of ipsilateral neck irradiation alone is the risk of failing in the base of tongue and contralateral neck.

Though side effects are less with unilateral neck irradiation that is not specifically directed at mucosal sites, xerostomia, mucosal atrophy, poor healing capacity in the radiated field, osteoradionecrosis, dysphagia and other dysfunction related to soft tissue fibrosis, and secondary malignancies, are all known late sequelae of radiation although less common and of lower intensity when using modern radiotherapy techniques (121).

A second approach for patients remaining in the NCUP category with low volume neck disease would be to start with neck dissection, which can be unilateral or bilateral (9, 104, 112, 120). This approach, which is the preferred approach under the NCCN guidelines (9) is advocated based on avoiding or reducing radiation, thereby lessening the risk of late toxicities, and obtaining more staging information. Neck dissection has its own morbidities, of which the most notable would be the risk of shoulder dysfunction related to manipulation of cranial nerve XI, which is usually transient.

If the patient and physician opt for neck dissection, and the final pathology report indicates a single involved lymph node, less than 3 centimeters in dimension, with low risk histopathological features (i.e. no extranodal extension, adequate number of dissected nodes available for histopatologic examination), current guidelines suggest that the patient can be closely observed with serial examination of the upper aerodigestive tract and serial fiberoptic office naso-pharyngo-laryngoscopy (9, 112). We expect a small percentage of these patients to reveal primary sites on long term follow-up, but no high level randomized data indicates the risk level involved in this choice. In some centers, such patients will be offered a postoperative dose of radiation, with or without a moderate radiation treatment to the highest risk mucosal surfaces, which can be restricted to the oropharynx for the HPV positive patients. This latter approach would still have significant reductions in dose and volume relative to primary radiation without neck dissection and would be standard for patients with multiple nodes or nodes larger than 3 centimeters. Multiple trials are ongoing studying HPV related cancers, and further de-escalations in treatment are being considered. Postoperative radiation in low to intermediate risk patients can involve doses of 44 to 63 Gy, with higher doses for areas with high risk pathological features. Extranodal extension, in particular, leads to an indication for adding chemotherapy to post operative radiotherapy (2, 9).

For advanced disease in the neck, surgery followed by radiation or combination of concurrent chemotherapy and radiation are the major options. For the HPV-related cancer, the latter will likely be recommended. For HPV negative cancer in an advanced state, if a reasonable resection can be devised to precede radiation, this may be preferred, but chemoradiation protocols that seek to avoid the dysfunction associated with radical neck dissection or extended radical neck dissection can also be offered. Strojan et al. (2) recommended surgery followed by radiation or concurrent chemoradiation followed by neck dissection in case of residual disease, and this was the algorithm typically applied to HPV-negative cancers, with documented improvements in survival when surgery was included in the initial treatment plan (2, 122). However, planned neck dissection in all patients, is no longer recommended. Salvage neck dissection is performed in patients with documented persistent neck disease, or residual adenopathy highly suspicious for viable cancer.

**Figure 2:**
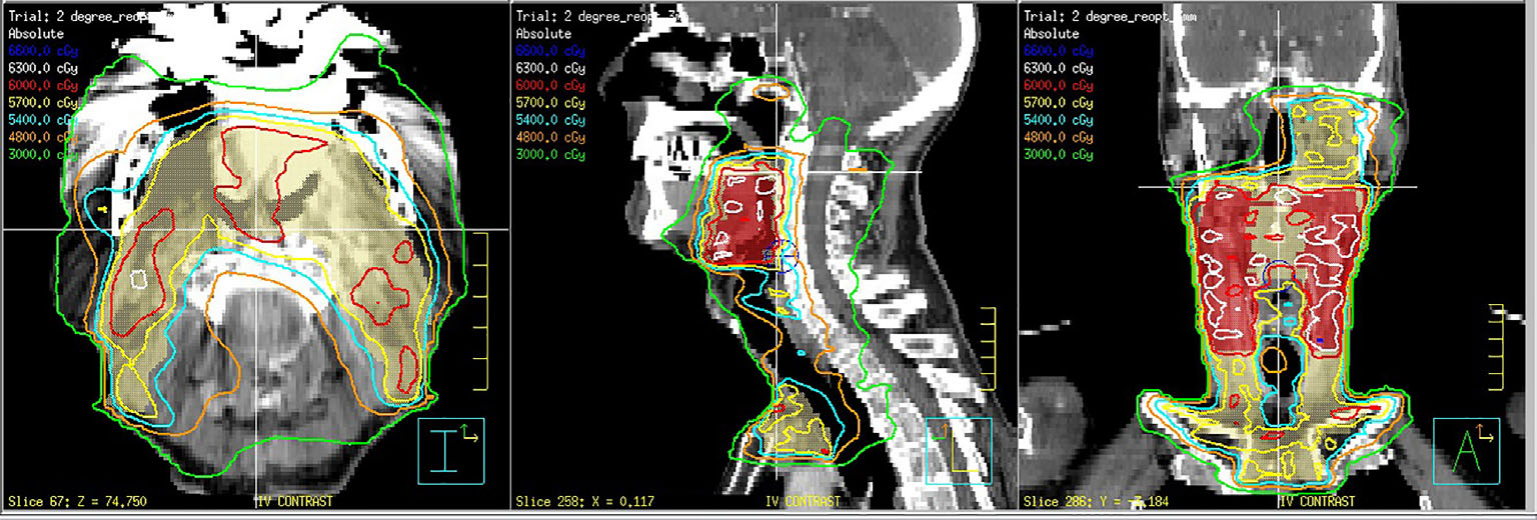
73-year-old man with an human papilloma virus (HPV) 16 positive squamous cell carcinoma from an unknown primary site with bilateral positive level 2 lymph nodes. The patient was treated with deintensified intensity modulated radiotherapy (IMRT) to 60 Gy in 30 once daily fractions to the oropharynx and bilateral positive nodes and 54 Gy in 30 fractions to the retropharyngeal nodes on the side with bulkier neck disease and levels 3 and 4 bilaterally. Concomitant weekly cisplatin 30 mg/M2 was administered. The patient tolerated treatment well and has remained disease free for 4.5 years without complications.

With HPV-related cancer, chemoradiation alone leads to high locoregional control rates, and the modern algorithm would be to treat advanced disease with chemoradiation protocols, usually including intravenous cisplatin in patients who can tolerate this agent and perform a PET CT at 3 to 6 months after treatment, followed by close clinical observation if deemed negative (9). Radiation dose is generally 70 Gy, 2 Gy per fraction to the grossly involved neck, and 50-60 Gy to mucosal sites at risk for harboring an occult primary, with increased doses of 60-66 Gy directed at highest risk sites (9) (**Figure 2**).

If a midline primary site of origin, i.e. base of tongue, cannot be ruled out, most radiation oncologists will prefer to treat neck bilaterally, as coming back to radiate later is usually difficult. Bilateral neck irradiation yielded non-significant improvements in mucosal and cervical disease control, albeit with significantly increased morbidity (123).

The functional differences between avoiding radiation to the mucosa, using limited fields to the oropharynx, and full mucosal irradiation to the nasopharynx, oropharynx, hypopharynx, and larynx, are significant. The latter, which historically represented the traditional treatment for NCUP is now occasionally used for HPV negative cases, mainly in patients with uncommon presentations such as level IV disease without positive nodes in level II and III. Many radiation oncologists prefer to treat the oropharynx, retropharyngeal nodes, and both sides of the neck. Enlarging the target volume to tightly include the nasopharynx may be considered and does not significantly increase morbidity. Routine inclusion of the larynx and hypopharynx is unlikely to encompass the NCUP and, thus, it does not compromise outcome but significantly increases morbidity and should be avoided if possible (124).

## Conclusions

Modern management of metastatic neck cancer with an unknown primary site requires proper recognition of the typical clinical presentation, and avoidance of diagnostic pitfalls that can lead to inappropriate interventions. Patients should be imaged with CT or MRI, and PET-CT, and receive a complete examination of the mucosal surfaces of the upper aerodigestive tract, preferably enhanced by the use of NBI. Ultrasound guided fine needle aspiration should be used for initial tissue diagnosis, but eventually histologic biopsy should be obtained, preferably from the occult primary site. HPV status, other immunohistochemical stains, and even next generation gene sequencing can guide us to probable primary sites. Tonsillectomy and lingual tonsillectomy may be indicated to identify a possible primary site.

Decisions regarding therapy are based on the primary tumor site, if identified, the stage of the neck disease, and the HPV/EBV status of the tumor. Neck dissection can be used to reduce radiation dosage in these patients and acquire increased histopathological risk stratification. We may be at the limit of what can be achieved by surgical technologies in terms of maximizing the rate of identification of primary tumors. Gene microarrays, and other molecular technologies, applied to at risk mucosal sites, may be the next step in identifying primary sites to target for treatment.

Both the diagnostic and therapeutic guidelines regarding management of NCUP continue to vary between medical centers. Core biopsy, lingual tonsillectomy, surgical treatment, primary radiation, surgery followed by radiation, chemoradiation, and multiple other details we have discussed are all recommended at different frequencies by different groups, even sometimes within the same geographic region (125).

Since NCUP, though more frequent in the HPV era, remains an uncommon diagnosis, it is difficult to perform large trials that lead to consensus. We hope to support the goal of achieving a consensus regarding the proper evaluation, subclassification, and treatment of these patients.

## Author Contributions

FC: First author, wrote original draft, did bulk of literature review and the largest amount of revising and editing. JV: Substantial editing/literature review, and dramatically changed the orientation of the manuscript. JS: Substantial editing/literature review, and dramatically changed the orientation of the manuscript. AR: Substantial editing/literature review. CS: Substantial editing/literature review. LK: Substantial editing/literature review. JR: Substantial editing/literature review. KO: Substantial editing/literature review, particularly contributed to the organization of the manuscript. PS: First authored prior articles 7 years ago to which these follow up, oversaw process in general including treatment section, edited and reviewed literature. AM: Substantial editing/literature review. RT: Substantial editing/literature review. RB: Substantial editing/literature review. JC: Substantial editing/literature review. VP: Substantial editing/literature review. AS: Substantial editing/literature review. DH : Substantial editing/literature review. WM: Provided **Figure 2**, substantial editing/literature review. CP: Substantial editing/literature review. MH: Substantial editing/literature review. TR: Substantial editing/literature review. NT: Substantial editing/literature review. AS: Substantial editing/literature review. AC-P: Substantial editing/literature review. JL: Substantial editing/literature review. JH-P: Substantial editing/literature review/focused on pathology. AF: Oversaw and coordinated the entire process, reviewed the article and edited multiple times, reviewed literature. He also dramatically altered the orientation of the manuscript. All authors contributed to the article and approved the submitted version.

## Conflict of Interest

The authors declare that the research was conducted in the absence of any commercial or financial relationships that could be construed as a potential conflict of interest.
